# FirebrowseR: an R client to the Broad Institute’s Firehose Pipeline

**DOI:** 10.1093/database/baw160

**Published:** 2017-01-06

**Authors:** Mario Deng, Johannes Brägelmann, Ivan Kryukov, Nuno Saraiva-Agostinho, Sven Perner

**Affiliations:** 1 Pathology of the University Medical Center Schleswig-Holstein, Campus Luebeck and the Research Center Borstel, Leibniz Center for Medicine and Biosciences, 23538 Luebeck and 23845 Borstel, Germany; 2 Molecular Pathology & Department of Translational Genomics, University Hospital Cologne, Weyertal 115b, Cologne, 50931, Germany; 3 Department of Biochemistry and Molecular Biology and Alberta Children’s Hospital Research Institute Calgary Biochemistry and Molecular Biology Doctoral Program in Bioinformatics, University of Calgary, Cumming School of Medicine, Alberta, Canada; 4 Instituto de Medicina Molecular, Faculdade de Medicina, Universidade de Lisboa, 1649-028 Lisboa, Portugal and; 5 Departamento de Informática, Faculdade de Ciências, Universidade de Lisboa, 1749-016 Lisboa, Portugal

## Abstract

With its Firebrowse service (http://firebrowse.org/) the Broad Institute is making large-scale multi-platform omics data analysis results publicly available through a Representational State Transfer (REST) Application Programmable Interface (API). Querying this database through an API client from an arbitrary programming environment is an essential task, allowing other developers and researchers to focus on their analysis and avoid data wrangling. Hence, as a first result, we developed a workflow to automatically generate, test and deploy such clients for rapid response to API changes. Its underlying infrastructure, a combination of free and publicly available web services, facilitates the development of API clients. It decouples changes in server software from the client software by reacting to changes in the RESTful service and removing direct dependencies on a specific implementation of an API. As a second result, FirebrowseR, an R client to the Broad Institute’s RESTful Firehose Pipeline, is provided as a working example, which is built by the means of the presented workflow. The package’s features are demonstrated by an example analysis of cancer gene expression data.

**Database URL:** https://github.com/mariodeng/

## Background

Sharing data in the field of cancer research is a common task, where the method of transfer and the file type strongly depend on the needs and available infrastructure of the curator. For data sets with a low grade of complexity it is common to provide tab or comma separated text files (TSV or CSV, respectively), as done with the Variant Call Format (VCF) [see Danecek *et al*. (1) for a details] files, or just plain text files as done with the Sequence Alignment Map (SAM), described by Li *et al* (2). Although these files might be compressed into the Binary Alignment Map (BAM) to lower their file size, they still carry needless information when only a subset is requested. Also these data types carry an overhead in the form of duplicated entries, making them non optimal for permanent storage and distribution of information, although they are used widely and represent the current standard. To reduce the amount of data and preserving the information, files can be transformed into a more complex format, allowing a more memory efficient way of storage. A common approach as used by database management systems (DBMS), is to only persist unique atomic components and the structural information in the form of linked tables. For the distribution across the network data sets may be provided as single large objects (as with VCF or SAM/BAM files) or in a non-redundant way as done with databases. Apart from the file size advantages and disadvantages between both approaches concerning the underlying infrastructure and their ease of implementation have to be taken into account. For example, automation of downstream analysis requires a constant file layout as applications could break due to format changes. As there is no standard protocol for flat files, ensuring such framework conditions, this feature is provided by DBMS. A common way to overcome these problems is to use a RESTful web API. As an API itself is just an arbitrary interface to an application, REST defines how the machine–machine interaction is realized. Even though this concept was introduced by Fielding (3) in 2000, there still is no standard definition. Regarding web applications it is common practice to realize the REST implementation over the Hypertext Transfer Protocol (HTTP) verbs, as defined by Berners-Lee and Fielding et al. in version 1.0 and 1.1 (4, 5). With an API in place, the software interaction with the database through an API will not be affected by changes to the database. For web applications, the API receives a query as a customized Uniform Resource Locator (URL), and delivers its results in a structured format, such as JavaScript Object Notation (JSON) or TSV files. This has the advantage that changes to the database do not affect the communication of client and server, as the database output is mapped to the API. Updates and changes to the API itself are communicated within its definition.

As the amount of omics data sets generated is increasing rapidly the mining and analysis of these data sets is preferable done on the latest release, live access to the stored information is necessary. With Firehose (https://gdac.broadinstitute.org/) the Broad Institute provides such live access to results of its data analysis pipeline with 14 729 cancer disease cases, distributed over 38 types of cancer at the time of writing. To build a client, which is kept updated with the API itself, we developed a workflow which automatically generates and deploys such software clients. As a working example we present FirebrowseR, an R client that allows the direct querying of the Firehose API from within the R programming environment. Just as other R software packages, like rentrez by Winter et al. (6)—providing an R interface to the Entrez database (7), and GO.db by Carlson e*t al*. (8)—giving access to the Gene Ontology database (9), FirebrowseR can be integrated into existing analysis workflows. Using such API clients over the download of flat files has several advantages, including having the latest data available, making the process of data importing obsolete and avoiding data re-formatting, which often serves as an additional source of errors. FirebrowseR’s source code and documentation is almost entirely generated automatically using the most current Firehose API definition. Following updates of the API definition the FirebrowseR code is automatically re-generated, tested and deployed. It thereby ensures that FirebrowseR releases are always synchronized with the API without requiring any action from the programmer, despite from updating the package GitHub.

## Implementation

The main benefit of using an API to retrieve data is that its definition is made available online through the API itself. This definition is structured in a hierarchical order and can be traversed from the root (entry point) of this hierarchy. This entry point is called the base URL and can be found under http://firebrowse.org/api/api-docs/for the API of the Broad Institute’s Firehose Pipeline. Here, on the top level, three basic entries can be found (i) *apiVersion*, (ii) *apis* and (iii) *swaggerVersion*, where the *apis* entry is a set of further definitions listing all available sub-*API*s and (i) and (ii) are server specific meta information. Traversing the *apis* entry the entire API definition can be explored, providing the developer with all information required to communicate with the API, such as method names, parameters and the expected HTTP method (e.g. GET or POST).

Out of these definitions almost all of the source code needed to implement a fully functional client can be generated. To do so it is common to use a logic-less template in the mustache format (https://mustache.github.io/). This template provides a raw construct of an R function including all the comments, which will be rendered as the documentation when the final package is built. The template used to build the FirebrowseR package from the Firehose API definition is shown and discussed in the supplemental code listing 1. It is designed in the way that for each function, a list of all the function arguments is created, which are than validated and combined to a final HTTP request. Afterwards, a download manager than executes this generalized HTTP request. This way any function can be represented in the same form and only one central download manager is needed.

To now combine both, the API definition and the template into interpretable R source code, the complete API definition is recursively traversed and the corresponding R code is generated.

## Workflow

**Figure 1.**
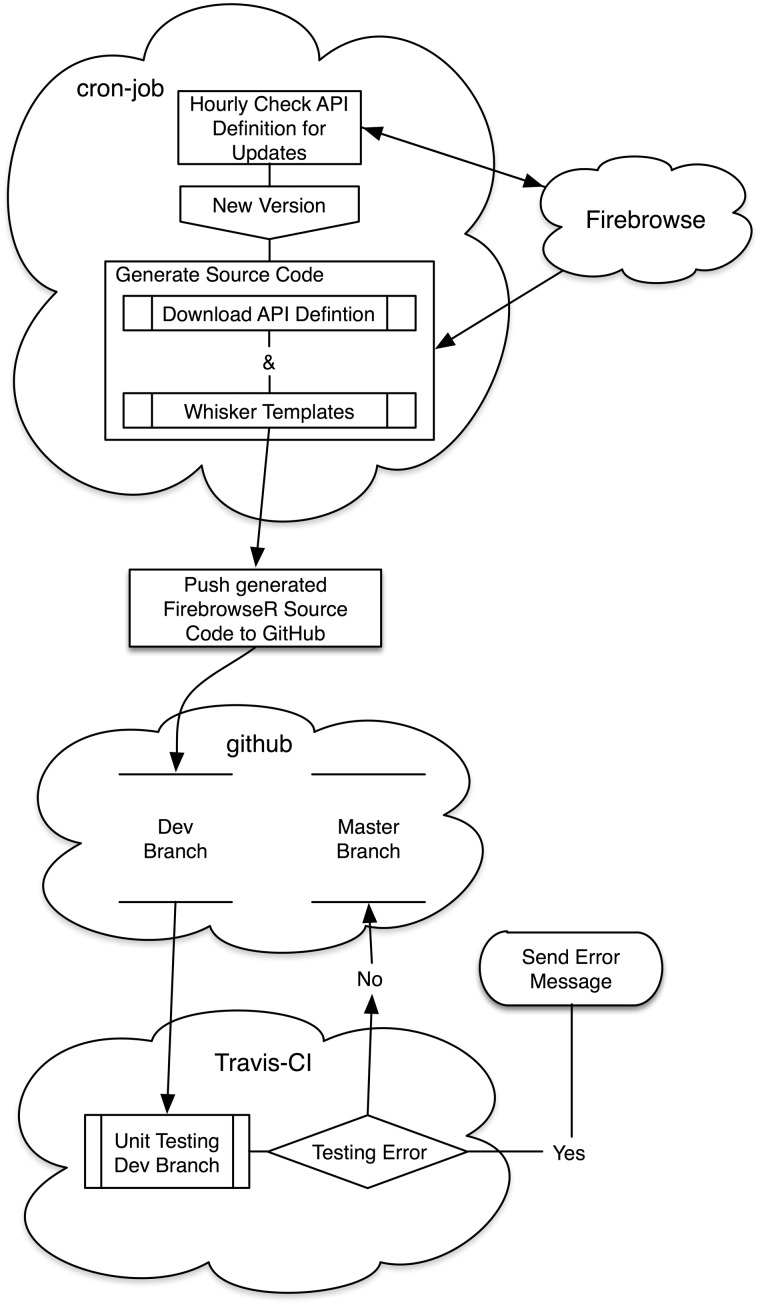
The complete system is composed of three web platforms. On cron-job.org the current API version is checked hourly and compared to the last one generated. If a new version is available, its definition is downloaded and the R source code is generated using whisker templates. The newly generated source code is then pushed to a developer branch on the second component, github.com. The third component, travisci.com, now applies pre-defined unit tests to the generated source on github’s developer branch. If no errors occur, the new R code is pushed into the repositories master branch. Otherwise the developer is notified via mail.

The entire workflow is a combination of free web services (Figure 1). The first problem to be tackled is to notice a change in the API definition. To do so, a cron-job (hosted on https://cron-job.org/) accesses the API hourly, parsing and comparing the APIs current version with the current master branch on GitHub (https://github.com/). If a new version is found, the source code is generated using R’s mustache implementation, whisker (please see technical details section for further information), and pushed to a developer branch on GitHub. This branch automatically spawns a unit test suite with upfront hand written unit tests (overall test coverage of 88% and 100% for API specific functions) at the Travis Continuous-Integration platform (https://travis-ci.org/), executing these tests are under Linux (Ubuntu LTS, release v12.04.5 as used by TravisCI at time of writing). If no errors occur, the newly generated code is pushed to FirebrowseRs master branch in GitHub and tested again, as errors could have occurred during pushing to another branch. If at any point a test does not pass, Travis-CIs event manager immediately contacts the developer team through email notifications.

## Results

Using the automated workflow all functions provided by the Broad Institute’s Firehose Pipeline are automatically generated and included into the FirebrowseR package. Therefore, the entire nomenclature of all functions, parameters, default and return values (including the return format) matches the API, simplifying the user’s transition between web API and the R programming environment. As described above, changes to the Firehose API trigger an automated update and predefined unit tests to FirebrowseR, keeping the package’s version in sync with the latest API.

One of the main differences in comparison of FirebrowseR to other existing software such as TCGA2STAT is that FirebrowseR implements exactly the same functions as the API, allowing users to narrow down their search on the query level by preselecting genes, samples or entities upfront (10). When using TCGA2STAT only the analyses type can be specified and an entire archive for the analyses type and cancer entity in question is downloaded from the Broad Institutes File Transfer Protocol (FTP) server, making it more suitable for exploratory analyses. This fact draws a clear line for the use cases of both software packages. While TCGA2STAT aims to provide its results as a basis for exploratory mass data analyses, FirebrowseR is considered a library to be used within other applications or hypotheses driven analyses where often a subset of analysis results for patients and genes is requested.

We are aware that besides TCGA2STAT other software packages exist, including TCGA-Assembler (11) and RTCGAToolbox (12). We did not include these packages into our discussion, as both packages (at time of writing) are outdated and have not received any updates recently. Further, neither package meets the current API definitions of the Broad Institutes Firehose service anymore, making the software unreliable.

## Example

**Figure 2.**
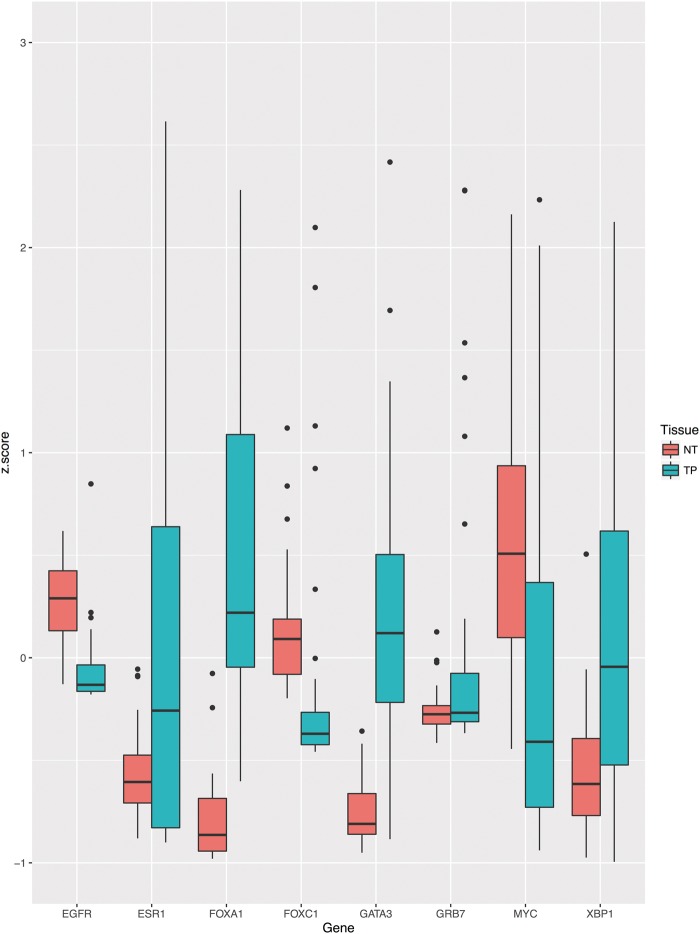
Boxplot indicating the expressions levels for tumor and adjacent normal tissue of deceased breast cancer patients. To provide clear example, well-known onco genes have been used to display potential differential expression.

In Code Listing 1, we give a short demonstration of FirebrowseR’s capabilities. As an example we created an analysis of breast cancer mRNA expression data for a user-defined set of candidate genes utilizing FirebrowseR only. After the package is installed and loaded (lines 1–5), the cohort’s metadata is downloaded and searched for the word ‘breast’, to determine the breast cancer cohort’s unique identifier (BRCA) (lines 6–9). Now clinical data is retrieved for all breast cancer patients using the *Samples.Clinical* function and only samples from deceased patients are kept for further analyses (lines 11–18). Utilizing the *Samples.mRNASeq* function mRNA expression data is download for determined samples and the genes ESR1, GATA3, XBP1, FOXA1, GRB7, EGFR, FOXC1 and MYC (lines 20–28). This set of expression values is further filtered, to keep just samples having tumor and adjacent normal tissue available (lines 30–35). As shown in Figure 2, ggplot2’s boxplot function is used to plot the mRNA expression levels for paired samples suffering from breast cancer disease, while expression levels are grouped by tissue (lines 37–41).

## Conclusion

Here we present a workflow that enables programmers to automatically generate clients to RESTful API interfaces, which are tested, updated and deployed automatically. As a result and working example of this workflow we introduced FirebrowseR, an R package for the interactive retrieval of data sets generated by the Broad Institutes Firehose Pipeline. With this capability of implementing the complete API interface, FirebrowseR is the perfect supplement to existing software, as it closes the niche between mass data and manual data retrieval. The complete project is hosted on GitHub (https://github.com/mariodeng/FirebrowseR) allowing every user to contribute and licensed under MIT license. User support is provided via the https://www.biostars.org platform, where we are actively following posts tagged with ‘FirebrowseR’.

The complete system is composed of three web platforms. On cron-job.org the current API version is checked hourly and compared to the last one generated. If a new version is available, its definition is downloaded and the R source code is generated using whisker templates. The newly generated source code is then pushed to a developer branch on the second component, github.com. The third component, travis-ci.com, now applies pre-defined unit tests to the generated source on github’s developer branch. If no errors occur, the new R code is pushed into the repositories master branch. Otherwise the developer is notified via mail.

Boxplot indicating the expressions levels for tumor (TP) and adjacent normal tissue (NT) of deceased breast cancer patients. To provide clear example, well known oncogenes have been used to display potential differential expression.

## Code listing 1

The source code used generated the boxplot for the samples of deceased breast cancer patients and the pre-selected gene of interest. The FirebrowseR package is used to download cohort, clinical and mRNA Expression data. Finally the expression values for paired normal and tumor samples are plotted using the ggplot2 library.

## Supplemental code listing legend 1

The template used to generate the R source code, which is then used by FirebrowseR. Variables being replaced by whisker are displayed in braces. For variables taking multiple parameters the *#* operator denotes an iteration over all available states a categorical function parameter can take. Further, negation can be taken into account by using the *^* sign.

## Technical details

FirebrowseR is implemented using the R programming language (https://www.r-project.org/) version 3.2.3 and tested using the testthat (v1.0.2) package (13) by Hadley Wickham. JSON parsing is done using the jsonlite (v1.0) package by Jeroen Ooms (14). The httr (v1.2.1) package by Hadley Wickham (https://cran.r-project.org/web/packages/httr/index.html) is used to determine HTML response. R’s Mustache implementation whisker (v0.3-2) (https://cran.r-project.org/web/packages/whisker/index.html) by Edwin de Jonge is utilized to fill the templates.

*Conflict of interest*. None declared.
